# Signaling Proteins Recruited to the Sperm Binding Site: Role of β-Catenin and Rho A

**DOI:** 10.3389/fcell.2022.886664

**Published:** 2022-05-13

**Authors:** Huizhen Wang, William H. Kinsey

**Affiliations:** Department of Anatomy & Cell Biology, University of Kansas School of Medicine, Kansa City, KS, United States

**Keywords:** oocyte, cortex, actin, fertilization, β-catenin, Rho A

## Abstract

Sperm interaction with the oocyte plasma membrane triggers a localized response in the mouse oocyte that leads to remodeling of oocyte surface as well as the underlying cortical actin layer. The recent demonstration that PTK2B is recruited and activated at the sperm binding site raised the possibility that multiple signaling events may be activated during this stage of fertilization. The present study demonstrated that β-catenin and Rho A were recruited to the cortex underlying bound/fused sperm. To determine whether sperm-oocyte contact was sufficient to initiate β-catenin recruitment, *Cd9*-null, and PTK2b-null oocytes were tested for the ability to recruit β-catenin to sperm binding sites. Both *Cd9* and *Ptk2b* ablation reduced β-catenin recruitment raising the possibility that PTK2B may act downstream of CD9 in the response to sperm binding/fusion. Further immunofluorescence study revealed that β-catenin co-localized with f-actin in the interstitial regions between actin layer fenestrae. Rho A, in contrast, was arranged underneath the actin layer in both the fenestra and the interstitial regions suggesting that they may play different roles in the oocyte.

## Introduction

The cortical actin layer is a major component of the oocyte cortex and undergoes important changes during oocyte maturation as described in a recent review (Santella et al., 2020). The mature mammalian oocyte exhibits a polarized structure based largely on the cortical actin layer which provides a framework for polarization based on actin layer thickness, microvillus abundance, and partitioning of the plasma membrane to arrange sperm binding proteins at the microvillar zone (Evans et al., 2000; Sun and Schatten, 2006). The cortical actin layer is unique in that it undergoes actin remodeling events initiated globally by Rho activity (Bement et al., 2015). In addition to global features, the cortical actin layer also undergoes highly localized reorganization at the site of sperm contact with the oocyte PM (Wang et al., 2017; Santella et al., 2020) which could play a role in sperm binding (Shalgi and Phillips, 1980; Runge et al., 2007; Jégou et al., 2011) and incorporation (Sutovsky et al., 1996; Kumakiri et al., 2003; Santella et al., 2018). Pharmacological disruption of the actin layer has been used to demonstrate the important role of the actin layer in fertilization and zygote development (Terada et al., 2000), however the specific mechanisms by which the oocyte cortex is maintained and the signals which induce its response to sperm-egg contact are poorly understood. One signaling modality that appears to function in actin remodeling at the sperm binding site involves protein tyrosine kinases in the SRC and FAK kinase families. Studies in invertebrate and vertebrate systems indicated that sperm-egg binding or fusion induced recruitment and activation of SRC family protein tyrosine kinases (Townley et al., 2009; Luo et al., 2010). Later studies in zebrafish and mice demonstrated recruitment of protein tyrosine kinase B (PTK2B) (Sharma and Kinsey, 2013) to the sperm binding site. PTK2B is a member of the focal adhesion kinase family and plays a role in actin-mediated events including lamellipodial activity (Okigaki et al., 2003), smooth muscle contraction (Tong et al., 2019), and neuronal cell process formation (Fan, 2018). In the mouse oocyte, functional studies demonstrate that PTK2B activity promotes actin remodeling events at the sperm binding site and facilitates sperm incorporation (Wang et al., 2017). The mechanism that transduces the signal provided by sperm-egg contact into PTK2B activation is not known, but CD9 expressed in the oocyte PM plays a major role in this process (Wang et al., 2021).

β-catenin has also been implicated in different aspects of fertilization, including sperm-egg binding and fusion (Takezawa et al., 2011). In addition, Rho-family GTPases have long been known to function in maintenance of the cortical actin layer (Bielak-Zmijewska et al., 2008) and recent studies demonstrate that Rho A induces actin polymerization in the form of repetitive waves within the starfish and frog oocyte cortex (Bement et al., 2015), leading ultimately to polar body extrusion (Varjabedian et al., 2018). Rho family members have also been implicated other fertilization events such as cortical granule exocytosis (Covián-Nares et al., 2004) and in sperm incorporation using the mouse oocyte model (Kumakiri et al., 2003). The above findings raise the possibility that β-catenin and Rho-family proteins might be recruited to the sperm binding site in manner similar to PTK2B and stimulate critical actin remodeling during fertilization. The objective of the present study was to examine the physical relationship of β-catenin and Rho A with the cortical actin layer and determine whether they are recruited to the sperm binding site as observed with the protein tyrosine kinase PTK2B. We then sought to determine whether CD9 expression is required for recruitment of β-catenin and Rho A at the sperm binding site. Another objective is based on our recent discovery of unique regions in the cortex where the actin layer is attenuated producing ‘fenestrae’ where the actin layer becomes thinner, potentially allowing cytoplasmic structures to gain close access to the plasma membrane (PM) (Wang and Kinsey, 2022). The presence of fenestrae suggests that the oocyte cortex has a complicated structure not previously recognized and here we have employed confocal immunofluorescence microscopy to determine how β-catenin and Rho A were arranged relative to these fenestrae.

## Methods

### Transgenic mice

*Ptk2b^−/−^* mice (Pfizer, New York, NY) were transferred from the laboratory of M. Pepper (Univ. of Washington, Seattle, WA) and genotyped as described (Lang et al., 2011). Mouse sperm null for *Cd9* ^−/−^ (*C57BL/6* ^Cd−9 tm1Osb^) (Miyado et al., 2000) (Center for Animal Resources and Development, Kumamoto University, Japan) were used to fertilize oocytes from C57BL/6 females (Jackson Laboratory, Bar Harbor, ME) to produce heterozygotes that would be fertile. Following blastocyst transfer, heterozygotes were maintained as breeding colonies to produce *Cd9* ^−/−^ females, with *Cd9* ^+/+^ littermates which were used as controls. Genotyping was performed as previously described (Miyado et al., 2008). B6D2F1 mice were obtained by crossing inbred C57BL/6NHsd females with inbred DBA/2NHsd males as described (Envigio, Indianapolis, IN). Animals were housed in a temperature and light cycle-controlled room and experiments were conducted in accordance with the ‘Guide for the Care and use of Laboratory Animals’ (Institute of Laboratory Animal Resources (U.S.) Committee on Care and Use of Laboratory Animals 1996; National Research Council (U.S.) 2011). Experimental procedures were approved by the University of Kansas Medical Center IACUC committee.

### Gamete Handling and *in vitro* Fertilization

*In vivo* fertilization: B6D2F1 females 4–5 weeks of age were stimulated with 5 IU of Pregnant Mares Serum Gonadotropin (PMSG) (Sigma-Aldrich, St. Louis, MO), followed 48 h later by 5 IU of human Chorionic Gonadotropin (hCG) (Sigma-Aldrich). Mating was initiated by adding a male (minimum of 12 weeks of age) to a cage holding a female >5 weeks of age. The females were sacrificed at 30 min intervals between 15.5–17 h post-hCG for collection of oocytes representing different stages of fertilization.

*In vitro* fertilization: Oocytes were collected from females 4–5 weeks of age stimulated with 5 IU of PMSG (Sigma-Aldrich, St. Louis, MO) followed 48 h later by 5 IU human Chorionic Gonadotropin (hCG) (Sigma-Aldrich). Cumulus-oocyte complexes (COCs) were collected at 15–16 h post-hCG, washed in Flushing and Handling Medium (Specialty Media Inc., Phillipsburgh, N.J.) containing 4 mg/ml BSA (FHM-BSA) (Sigma-Aldrich), then cumulus cells were removed by treatment with 30 IU/ml hyaluronidase (Sigma-Aldrich) for 10 min. Zona-removal was performed by exposing oocytes to two consecutive 100ul drops of Acid Tyrode’s (Sigma-Aldrich), then transferred to 1 ml of FHM-BSA for pH neutralization. Zona-free eggs were washed into CARD medium United States (KYD-005-EX, Cosmo Bio United States (Carlsbad, CA)) and allowed to recover under oil for 1 h. B6D2F1 males were capacitated by incubating cauda epididymal sperm in FertiUp medium (KYD-005-EX, Cosmo Bio United States (Carlsbad, CA)) at 37°C and 5% CO_2_ in air under mineral oil for 1 h Capacitated sperm were added to oocytes at a final concentration of 2 × 10^4^/ml and incubated under oil at 37°C and 5% CO2 for 20 min to allow *in vitro* fertilization.

### Oocyte Fixation and Immunolabeling

Fixation conditions designed to study actin layer fenestrae used oocytes fertilized *in vivo* to minimize culture or handling induced artifacts. Oviducts were removed from virgin or mated females and submerged in fixative containing 2% formaldehyde with 1% saturated picric acid in PBS. The ampullae were dissected free of remaining oviductal tissue then cut to release cumulus-enclosed oocytes into the fixative. After 1 h incubation at 25°C, cumulus enclosed oocytes were transferred to a refrigerator for an additional 20 h of prolonged fixation at 4°C based on an earlier method (Morency et al., 2011). Zona free oocytes fertilized *in vitro* were fixed in 2% formaldehyde with 1% saturated picric acid in PBS containing 40 uM phenylarsine oxide (Sigma-Aldrich) and 100 uM sodium ortho-vanadate (ThermoFisher Scientific, Waltham, MA) as phosphatase inhibitors. After fixation, oocytes were incubated in blocking buffer consisting of PBS with 0.1% triton X-100 (Sigma-Aldrich) and 3 mg/ml BSA for 30 min, then incubated with primary antibodies diluted 1/100 in blocking buffer overnight. Primary antibodies used include rabbit anti-human non-phospho (Active) β−catenin ((Ser33/37/Thr41, Cat # 8814), Cell Signaling, Danvers, MA), rabbit anti- β−catenin (epitope different from the non-phosphorylated antibody from Cell Signaling) [(Cat # BD610153) Becton, Dickinson, and Co. Franklin Lakes, NJ], rabbit anti-human RHOA (Cat. # 10749-1-AP, Proteintech, Rosemont, IL). For detection of cortical granules, FITC conjugated -Lens culinaris agglutinin [(LCA) Cat. # L32475, Invitrogen San Diego, CA] was used at 5 ug/ml. The specificity of the non-phospho (Active) β−catenin (Ser33/37/Thr41) antibody was tested by addition of a synthetic peptide (DSGIHSGATTT aa32-aa42) from GenScript (Piscataway NJ) as a competitive inhibitor of the primary antibody at 1:100 excess (Supplementary Figure S1). Specificity of the RHOA antibody was tested by addition of RHOA fusion peptide (Cat. # AG1141 Proteintech, Rosemont, IL) as a competitive inhibitor of the primary antibody at 1:100 excess (Supplementary Figure S1). Secondary labelling was done for 1 h in blocking buffer containing Alexa Fluor 488 goat anti-rabbit IgG (In Vitrogen, Waltham, MA (A11008)) or Alexa Fluor 555 goat anti-rat (Invitrogen, (A11006)), diluted 1:100 in blocking buffer. Alexa Fluor 568-phalloidin (Invitrogen) (2 U/ml), and Hoechst 33342 (0.1 mg/ml) were included with all secondary antibodies and labelled oocytes were transferred to glycerol mounting medium (Abcam, AB188804) prior to imaging.

### Live Cell Imaging

Live cell imaging was performed on cumulus-enclosed oocytes recovered from mated females. Cumulus-enclosed oocytes were treated with hyaluronidase (30IU/ml) for 10 min to remove cumulus cells, then incubated in 500ul of KSOM-BSA for 30 min at 37°C and 5% CO2 in a humidified incubator. Then 0.5 µl of SiR-actin (Cytoskeleton Inc. Denver, CO) (1 mM) was added to achieve a final concentration of 1 µM and the oocytes were returned to the incubator for an additional 30 min. Oocytes were then washed in KSOM-BSA and transferred to a poly A-lysine (Sigma-Aldrich) treated Delta-TPG plate (Bioptics, Butler, PA) containing a 10ul drop of KSOM-BSA containing Hoechst 33342 (1ug/ml) and covered with pre-equilibrated mineral oil. The plate was mounted in a Chamlide (Quorum Technology, Inc. Guelph, ON, Canada) TC-L stage top environmental chamber to maintain temperature at 37°C and a CO_2_ level of 5%. Imaging was performed with a 640 nm laser to detect the actin fenestrae labelled with SiR-actin.

### Confocal Microscopy

Confocal microscopy was performed with a Nikon A1R microscope, using sequential scans with constant beam intensity. Images were recorded from Z-planes containing one or more sperm heads bound to the oocyte surface. Fluorescence associated with sperm binding sites at the oocyte plasma membrane and associated cortical actin layer was quantified by linescan analysis in the green channel to detect anti-β-catenin and in the red channel to detect Alexa Fluor 568-phalloidin as a measure of f-actin, and in the blue channel to detect Hoechst 33342 labeled chromatin in sperm that were bound to or fused with the PM. Graphical presentation was used to identify inflection points in the blue channel which defined the edges of each sperm binding site and allowed creation of a baseline. Fluorescence above baseline in the red and green channels within the sperm binding site was integrated and divided by mean fluorescence above baseline in adjacent cortex to each side of the binding site to define the relative fluorescence intensity of each sperm binding site as described (Wang et al., 2017). The integrated relative fluorescence intensity was used to provide a measure of β-catenin-associated fluorescence at each sperm binding site.

STimulated Emission Depletion (STED) microscopy was performed with a Leica TCS SP8X STED ONE microscope.

### Statistics

The statistical significance of differences in the median relative integrated fluorescence intensity reporting β-catenin was demonstrated using the Kruskal-Wallis One Way Analysis of Variance on Ranks (Systat Software, San Jose, CA).

## Results

### Recruitment of β-catenin and RhoA to the Sperm Binding Site

**FIGURE 1:**
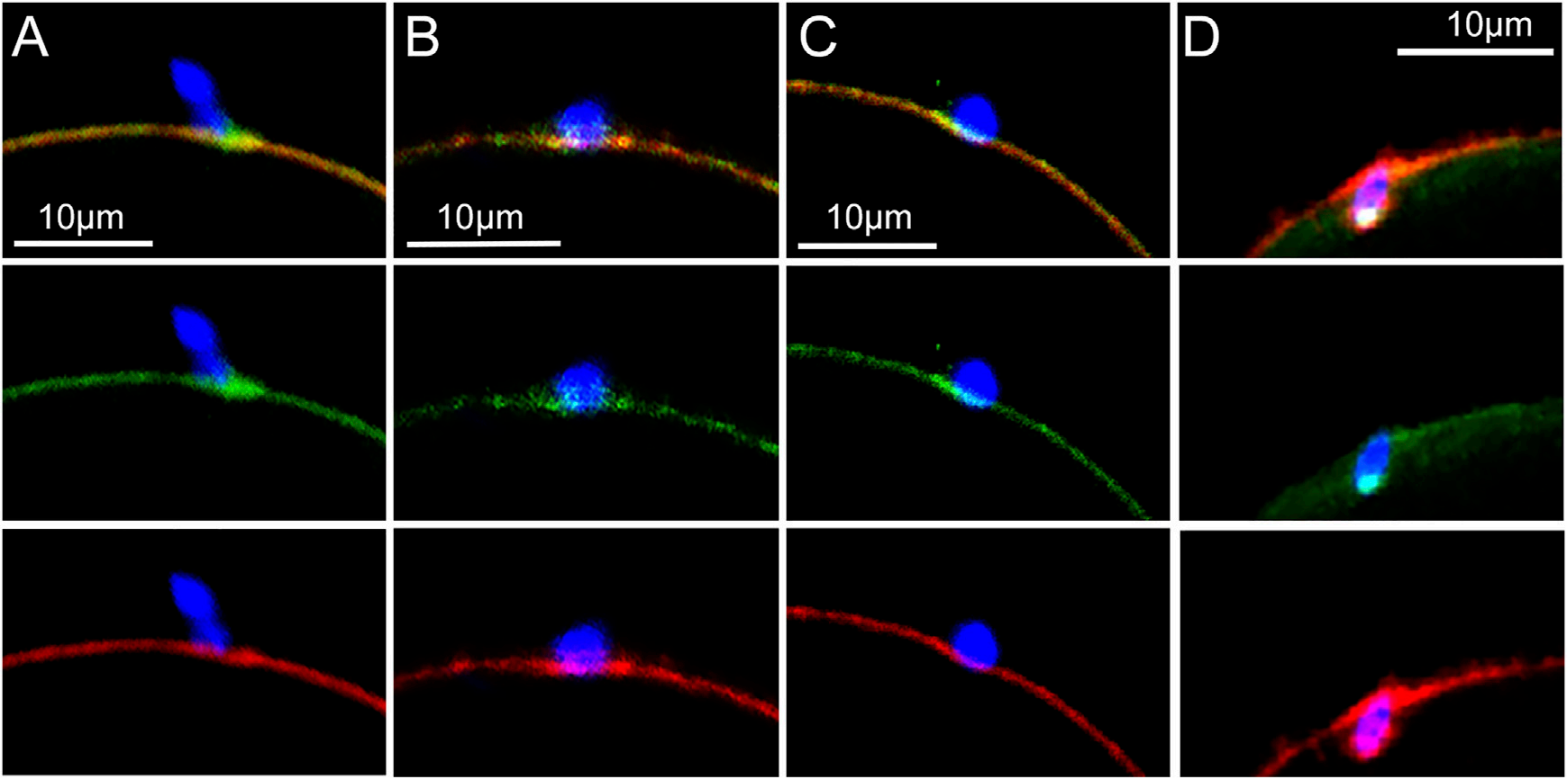
Localization of β-catenin at the sperm binding site.Oocytes fixed at different stages of fertilization were labeled with anti- β-catenin (green) and Alexa Fluor-568 phalloidin (red) as well as Hoechst 33342 (blue). **(A)** Recently bound sperm. **(B)** bound sperm with microvilli exhibiting β-catenin labelling. **(C)** β-catenin plaque colocalizing with the actin layer. **(D)** sperm undergoing incorporation into the ooplasm with a single bright patch of β-catenin remaining. Images were selected from 30 oocytes recovered from 4 females. Magnification is indicated by the bar.

*In vitro* fertilization of mature, zona-free oocytes was performed to determine whether β-catenin and Rho A became concentrated at sperm binding sites during fertilization. Under the conditions described in Materials and Methods, oocytes fixed at 20 min post insemination (mpi) captured examples (n = 30 oocytes) where sperm had bound to or fused with the oocyte PM. Oocytes with sperm that had minimal contact with the oocyte often exhibited accumulation of β-catenin (green) within the f-actin layer (red) at the sperm contact site (Figure 1A). Elongated cell processes, presumably microvilli which formed at the sperm contact site exhibited strong β-catenin labelling (Figure 1B). As sperm established broader contact with the oocyte surface, β-catenin became concentrated as a plaque within the cortical actin layer underlying the sperm head (Figure 1C). During the early stage of sperm incorporation, the β-catenin plaque was dispersed (Figure 1D), often leaving a small, bright region of β-catenin in close proximity to the sperm head. The antibody used to detect β-catenin here was specific for the de-phosphorylated (Ser33/37 &Thr41) form of β-catenin which is more stable in live cells, however similar images were obtained using an antibody against a different epitope that is not specific for the de-phosphorylated form (Materials and Methods).

**FIGURE 2:**
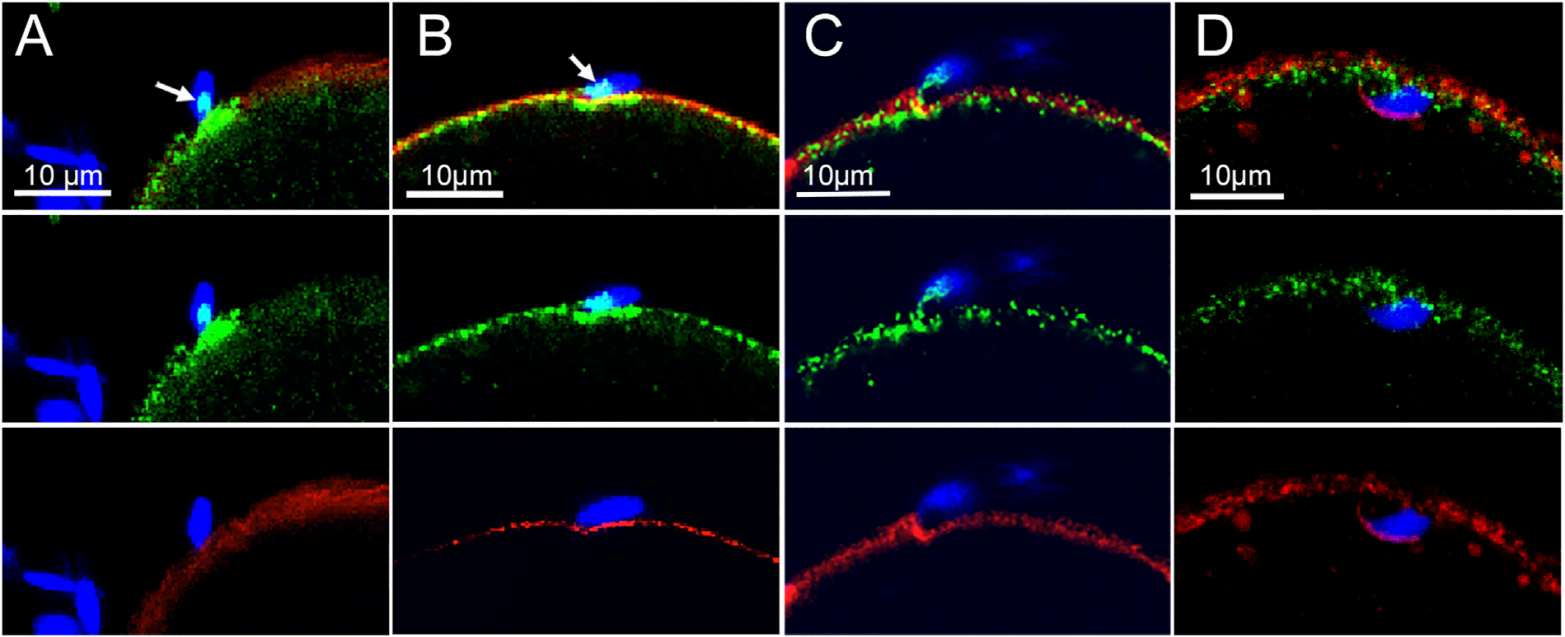
Localization of Rho A at the sperm binding site.Oocytes fixed at different stages of fertilization were labeled with anti- Rho A (green) and Alexa Fluor-568 phalloidin (red) as well as Hoechst 33342 (blue). **(A)** recently bound sperm with a Rho A patch on the sperm head (arow) and a plaque of oocyte-derived Rho A underneath. **(B)** oocyte Rho A plaque underling the actin layer at the sperm binding site (arrow indicates a patch of sperm Rho A). **(C)** invagination of the cortical actin layer with associated Rho A foci **(D)** incorporated sperm with few remaining Rho A foci. Images were selected from 24 oocytes recovered from 3 females. Magnification is indicated by the bar.

The distribution of Rho A (green) at the sperm binding site (n = 24 oocytes) was complicated by the fact that the sperm acrosomal region contains significant Rho A (Ducummon and Berger, 2006; Reyes-Miguel et al., 2020). However, depending on the angle of the image plane, Rho A concentrated in the underlying oocyte cortex could be distinguished from the Rho A bound to sperm (Figures 2A,B). As surface contact between sperm and oocyte increased, oocyte Rho A became concentrated into a plaque in the underlying cortex (Figure 2B). The Rho A plaque appeared similar to that formed by β-catenin with the exception that Rho A was localized underneath the cytoplasmic surface of the f-actin layer (red) instead of co-localizing with f-actin as occurs with β-catenin. As the oocyte cortex began to invaginate Rho A labeling became somewhat dispersed (Figure 2C) and as the sperm began to be incorporated into the ooplasm, Rho A diminished greatly leaving only a few small Rho A foci (Figure 2D).

### Effect of *Ptk2b* and CD9 Ablation on β-catenin

The finding that PTK2B activation at the sperm binding site plays a role in actin remodeling at that site (Wang et al., 2017) raises the possibility that PTK2B may stimulate recruitment of other signaling proteins such as β-catenin. Therefore, we tested the effect of PTK2B ablation on β-catenin recruitment in response to interaction with sperm during zona-free IVF. The relative amount (relative to adjacent cortex) of β-catenin-related fluorescence at sperm binding sites was quantified and compared to that in *wt* oocytes (Materials and Methods). Oocytes from *Ptk2b* ^*−/−*^ females exhibited a partial, but significant decline in β-catenin recruitment as seen in Table 1. Since we had earlier learned that PTK2B activation at the sperm binding site is heavily dependent on the expression of the sperm binding protein CD9 (Wang et al., 2021), we tested the possibility that β-catenin may also require CD9 expression in order to respond to sperm binding. Quantification of the relative β-catenin-related fluorescence at sperm binding sites in *wt* and *Cd9^−/−^* oocytes revealed that the *Cd9* ^*−/−*^ *oocytes* exhibited very little recruitment of β-catenin compared to the control group (Table 1).

**TABLE 1:** Effect of *Ptk2b* or *Cd9* ablation on β-catenin recruitment to sperm binding sites.

Genotype		# Oocytes	# Binding sites	Median Integrated
Sperm	Oocyte		Analyzed	Fluorescence
*wt*	C57BL/6	26	118	64% ± 5.0%
*wt*	*Ptk2b* ^*−/−*^	23	103	34% ± 6.4%*
*wt*	*Cd9* ^*−/−*^	15	122	16% ± 3.3%*

The integrated relative fluorescence intensity of β-catenin at sperm binding sites was determined and the median values are presented +/− SEM. Data from the control C57BL/6 oocytes includes 4 replicates (13 females); data from *ptk2b* ^-/-^ oocytes includes 3 replicates (8 females); and data from the *Cd9* ^-/-^ oocytes includes 3 replicates, (7 females). (*) indicates the median was significantly different from that of the control (C57BL/6) oocytes (P = <0.001).

### Structure of Actin Layer Fenestrae

**FIGURE 3:**
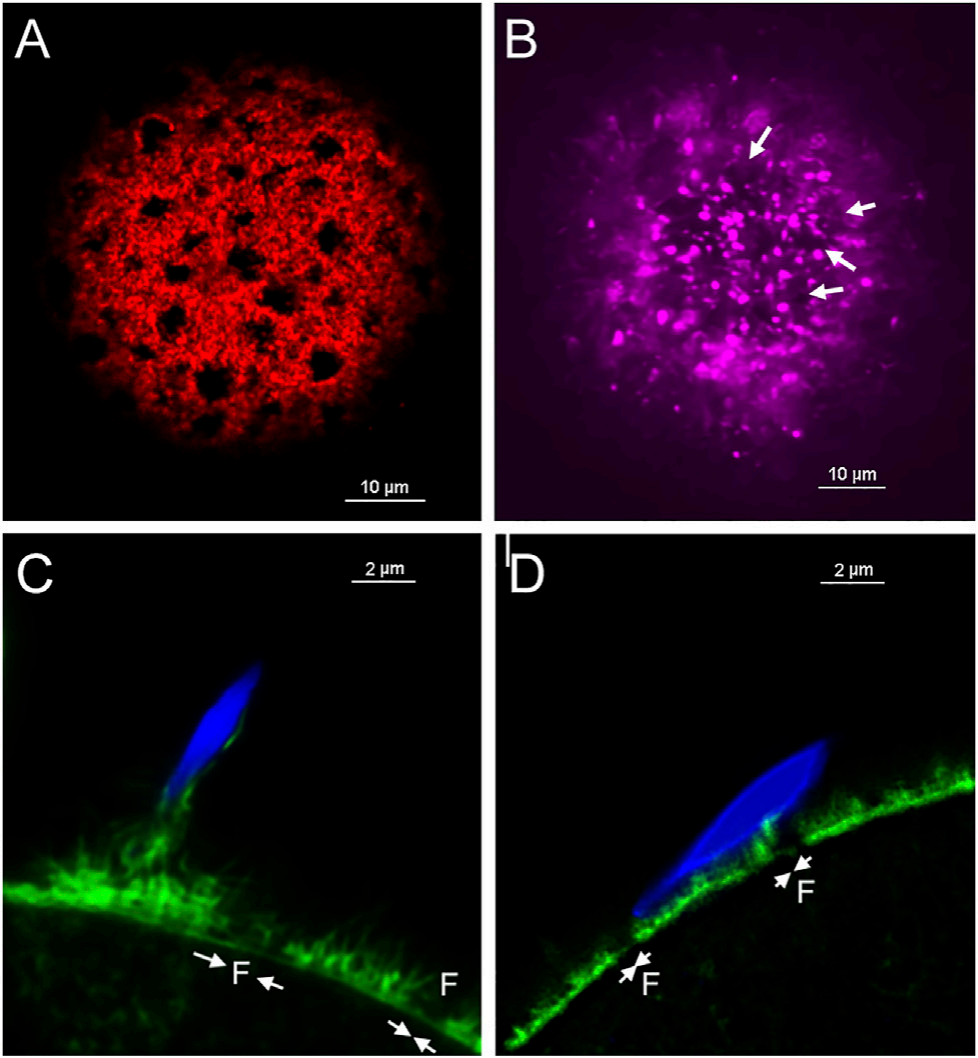
Fixed and Live cell imaging of fenestrae in the cortical actin layer.Oocytes with bound/fused sperm from mated females were fixed and labelled with alexa-Fluor 568 **(A)**. Live oocytes with partially incorporated sperm were selected from mated females and labeled with SiR-actin as described in Methods, (arrows indicate the position of some of the fenestrae) **(B)**. Oocytes fertilized *in vitro* (n = 19) were fixed and labelled with alexa-Fluor 568 phalloidin then imaged by STED microscopy to observe the distribution of microvilli at the sperm binding site. Examples include a supernumerary sperm bound to an oocyte already containing an incorporated sperm **(C)**, and an unfertilized (no incorporated sperm found) oocyte with a bound or fusing sperm **(D)**. Arrows indicate the presence of fenestrae. Magnification is indicated by the bars.

β-catenin was earlier shown to be localized in the cortical actin layer of mouse oocytes (Takezawa et al., 2011), however our recent discovery of fenestrae in the cortical actin layer (Wang and Kinsey, 2022) prompted us to revisit the arrangement of the oocyte cortex. Standard confocal imaging of the actin layer in fertilized oocytes recovered from mated females and subjected to prolonged formalin fixation (Materials and Methods) then labelled with Alexa Fluor 568-phalloidin reveals the presence of fenestrae as dark (very low actin fluorescence), irregular regions which are surrounded by highly fluorescent interstitial regions (red) where the actin layer is much thicker (Figure 3A). A similar pattern was observed in live fertilized oocytes labelled with SiR-actin (purple) (Figure 3B) indicating that the fenestrae were not artifacts of fixation. Examination of 31 oocytes prepared with prolonged fixation revealed variation in the size and shape of fenestrae (Supplementary Figure S2), but we have not found consistent changes in response to fertilization. The use of STED microscopy revealed more detail regarding differences in actin structure (green) between fenestrae and interstitial regions. In most oocytes examined, microvilli were most abundant in the interstitial regions of cortex in both fertilized and unfertilized oocytes (Figures 3C,D). We also observe that bound sperm could potentially make contact with both an interstitial region and a fenestra (Figure 3C) (see discussion). Another important feature of the oocyte cortex is the associated cortical granules that are secreted in response to fertilization. Analysis of the distribution of cortical granules in oocytes from virgin females revealed that cortical granules (green) were, for the most part, localized under interstitial regions (red) or at the boundary between fenestrae and interstitial regions (Supplementary Figure S3A). In oocytes from mated females, many cortical granules had migrated into fenestrae while others appeared on the outer surface of the interstitial actin layer (Supplementary Figures S3B,C).

### β-catenin & RhoA Localization Relative to Actin Layer Fenestrae

**FIGURE 4:**
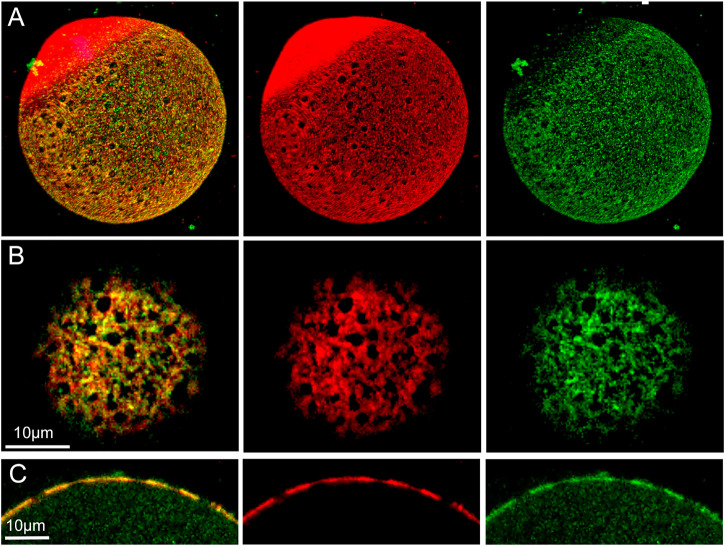
Distribution of f-actin and β-catenin in the oocyte cortex.MII oocyte from a virgin female was treated by prolonged fixation and labeled with anti- β-catenin (green) and Alexa Fluor-568 phalloidin (red) to label f-actin, as well as Hoechst 33342 (blue). Z-stack images viewed in volume mode **(A)**, a tangential image **(B)**, and an equatorial image **(C)** reveal fenestrae appearing as holes in the cortical actin layer **(A,B)** or as regions of thinner f-actin in the equatorial view **(C)**. Images were selected from 10 oocytes from three females. Magnification is indicated by the bar.

In order to discover how β-catenin is distributed relative to actin fenestrae, we performed standard confocal immunofluorescence on oocytes fixed for 21 h. The extended fixation procedure preserved numerous fenestrae present in the microvillar zone of the cortex (red) (Figure 4A) which are seen at higher magnification in a tangential view (Figure 4B). 3D reconstruction confirms the polarized distribution of β-catenin (green) (Figure 4A). Images taken through the equator of the oocyte demonstrate that fenestrae represent regions where the actin layer is attenuated (Figure 4C) while the interstitial regions between fenestrae retain normal thickness. Careful examination of the distribution of β-catenin in tangential focal planes demonstrates that β-catenin is concentrated at the interstitial regions between fenestrae and is less abundant within the fenestrae themselves (Figures 4B,C). Equatorial views (Figure 4C) also reveal that β-catenin was mostly co-localized with the f-actin layer, although small accumulations were sometimes observed superficial to the actin layer.

**FIGURE 5:**
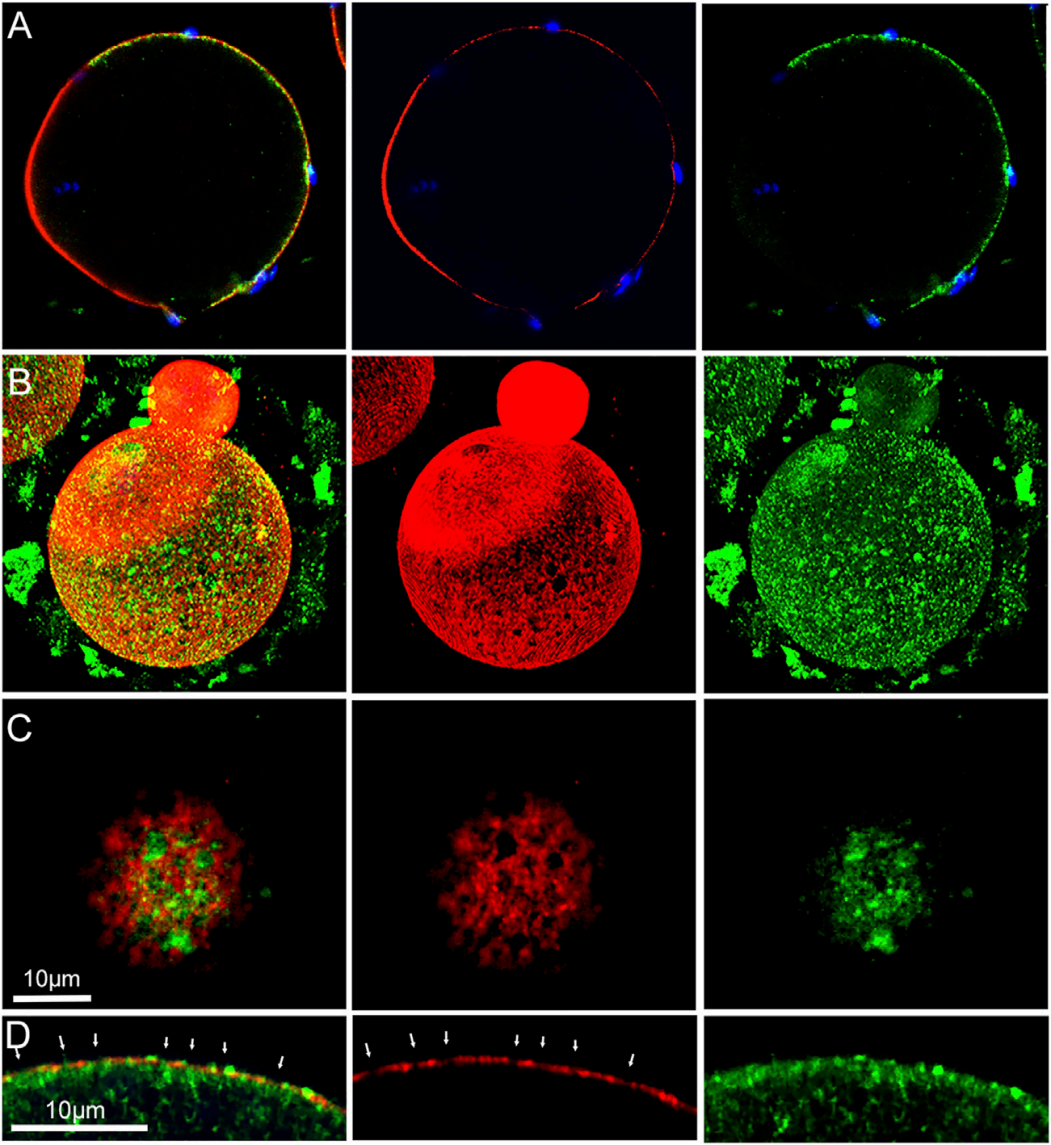
Distribution of f-actin and Rho A in the oocyte cortex.MII oocyte from a virgin female fixed and labeled with anti- Rho A (green) and Alexa Fluor-568 phalloidin (red) as well as Hoechst 33342 (blue) showing the polarized localization of Rho A **(A)**. Oocyte from a mated female was treated by prolonged fixation and labeled as above. Z-stack images viewed in volume mode **(B)**, or a tangential image reveal the association of fenestrae with Rho A aggregates **(C)**. An equatorial image reveals the position of Rho A aggregates under some fenestrae **(D)**. Arrows indicate the position of fenestrae with underlying Rho A aggregates and occasional Rho A foci (bright green) overlying f-actin interstitial regions. Images were selected from 12 oocytes recovered from 3 females. Magnification is indicated by the bar.

Examination of oocytes labelled with anti-Rho A (green) demonstrate that it is also arranged in a polarized fashion, and appears in the form of irregular aggregates underneath the actin layer (red) (Figure 5A). 3D and tangential and images demonstrate that Rho A is clearly present within fenestrae (Figures 5B,C). An equatorial view shows the irregular character of Rho A aggregates which are mostly localized under the inner surface of the actin layer, although small aggregates are also present on the outer surface (Figure 5D). Rho A aggregates are present underneath both interstitial regions and fenestrae (Figure 5D arrows). In equatorial images, it also appears that Rho A aggregates exhibit little colocalization with the actin layer.

## Discussion

The cortical actin layer in mammalian oocytes establishes a polarized cortex where PM components involved with fertilization are separated from the meiotic spindle. Examples include segregation of microvilli, cortical granules and cortical ER clusters to the microvillar zone. Sperm binding proteins such as CD9 are also concentrated in the microvillar zone (Runge et al., 2007) which likely promotes sperm binding to that region of the oocyte. Further evidence of polarization lies in the distribution of signaling proteins such as β-catenin and Rho A (Takezawa et al., 2011; Bement et al., 2015). In addition to these global specializations, the actin layer responds to sperm binding and fusion with a localized response, including extension of microvilli to enhance PM contact with the sperm head and formation of a wave-like PM protrusion (Satouh et al., 2012; Ravaux et al., 2018) that functions during sperm incorporation. As an initial step in understanding how these unique aspects of the actin layer are controlled, this study has examined the distribution of β-catenin and Rho A in the mature oocyte cortex, their recruitment to the sperm binding site, and the role of PTK2B and CD9 in this recruitment event.

### Recruitment of β-catenin and Rho A to the Sperm Binding Site

We demonstrate that once a sperm has bound to or fused with the oocyte PM, both β-catenin and Rho A are recruited to the sperm binding site within 20 min of insemination. β-catenin continued to be closely associated with f-actin within elongated microvillus structures typical of the sperm binding site. Unlike the recruitment of PTK2B at sperm binding sites (Wang et al., 2017) where many bound sperm elicit no response by the oocyte, β-catenin recruitment is visible at the majority of sperm binding sites. This could indicate that β-catenin recruitment is independent of or upstream of PTK2B recruitment. The association of β-catenin with structures involved in stabilizing the sperm (thickening of the actin layer and contact with microvilli) is consistent with the observation that β-catenin-*null* oocytes exhibit reduced sperm binding capability (Takezawa et al., 2011). Rho A recruited to the sperm binding site was not significantly involved in microvilli or other cell processes making contact with the sperm. As the extent of surface contact between sperm and oocyte increased, a ‘plaque’ like region of β-catenin formed within the underlying actin layer, while Rho A became concentrated underneath the actin layer plaque. It is interesting to compare the responses of β-catenin and Rho A to that of PTK2B at the sperm binding site. PTK2B is primarily recruited to the cytoplasmic side of the actin layer underlying a bound sperm (Wang 2017). But as PTK2B becomes activated (phosphorylated on Y^402^), the resulting PTK2B-Y^402^ was also observed within microvilli and larger cell processes in contact with the sperm (Wang et al., 2021). In contrast, Rho A remains localized to the cytoplasmic side of the actin layer plaque. Once the sperm is incorporated, PTK2B-PY^402^, β-catenin, and Rho A disperse into the ooplasm.

The similar behavior of β-catenin and PTK2B raises the possibility that both may be part of a pathway involved in actin remodeling at the sperm binding site. The findings that *Cd9* ablation suppresses both PTK2B activation (Wang et al., 2021) and β-catenin recruitment to the sperm binding site, suggests that such a pathway may exist. One might speculate that CD9 enables the oocyte to respond to sperm binding by activating PTK2B which, in turn, stabilizes β-catenin at the sperm binding site. The initial signal for such a pathway could occur as a result of sperm-oocyte binding or of gamete fusion and subsequent Ca^2+^ oscillations. CD9 is generally recognized to be required for sperm-egg fusion in mice (Miyado et al., 2000), so our observation that *Cd9* ^*−/−*^ oocytes are much less effective at recruiting β-catenin to sperm binding sites would imply a requirement for gamete fusion. Further studies blocking fusion by other techniques (Wang et al., 2017) or suppressing Ca^2+^ oscillations artificially would resolve this important question.

The mechanism by which PTK2B could influence β-catenin remains unknown, however, it has been proposed that PTK2B regulates phosphorylation of glycogen synthase kinase 3β (GSK3β) (Gao et al., 2015; Yang et al., 2015; Gao et al., 2019) resulting in β-catenin stabilization. Other studies have proposed that PTK2B can stabilize β-catenin directly by phosphorylation at Y^654^ (Gao et al., 2019).

### Features of Actin Layer Fenestrae

During the course of this study, we examined the positioning of cortical granules and microvilli relative to fenestrae in the actin layer. We found that cortical granules were rarely seen in fenestrae of unfertilized oocytes, but upon sperm binding or fusion, many of them migrated into fenestrae to establish close proximity to the plasma membrane while others appeared to penetrate interstitial regions directly. The distribution of microvilli in the actin layer was most easily observed by STED microscopy which revealed that in fenestrae, the ‘actin layer’ appeared as a relatively thin, flat layer with few microvilli. In contrast, the interstitial regions exhibited a thicker actin layer with accumulations of amorphous f-actin on its outer surface from which a dense array of microvilli extend. This arrangement has implications for sperm-egg interaction and potentially for gamete fusion as described below.

### Localization of β-catenin and Rho A

β-catenin is a signaling protein abundant in the oocyte cortex, co-localizing with the actin layer in the microvillar zone (Takezawa et al., 2011) and undergoing tyrosine phosphorylation at the MII stage (Ohsugi et al., 1999). Through the use of confocal immunofluorescence microscopy, we demonstrate that β-catenin is not uniformly distributed in the cortex but instead is concentrated in the interstitial regions between fenestrae while the fenestrae themselves exhibit low, near background levels of β-catenin. F-actin was found to co-localize with β-catenin as previously described (Takezawa et al., 2011), although we also observed significant β-catenin at the base of microvilli. Since the interstitial regions are rich in f-actin, microvilli, and β-catenin, it is interesting to speculate that they provide ideal sites for tethering E-cadherin/β-catenin complexes (Ohsugi et al., 1999) and potentially serve to transduce outside signals initiated by sperm binding or gamete fusion. In fenestrae, the paucity of microvilli and the low amount of β-catenin and actin may confer more flexibility to the overlying PM. Jegou and co-workers (Jégou et al., 2011) proposed that a flexible region of cytoskeleton could tightly conform to the sperm surface creating a strong “S” type adhesion where the two PMs could remain in direct contact long enough to allow fusion to occur. This hypothesis would suggest that sperm have a greater chance of successful fusion by binding to regions of oocyte PM overlying fenestrae rather than interstitial regions between fenestrae. In fact, the case may be that the oocyte cortex is designed to supply both environments. As seen in Figures 3A,D, sperm may establish stable binding through contact with an interstitial region, yet still overlap a fenestra where the actin layer is thinner and flat. While speculative, there is reason to compare the cortical stiffness, abundance of sperm binding proteins, and frequency of sperm-egg fusion events in PM overlying actin layer fenestrae vs. interstitial regions.

Rho A is expressed mainly in the cortex of sea urchin and porcine oocytes (Covián-Nares et al., 2004; Zhang et al., 2014), and here we present confocal images demonstrating Rho A localization to the cytoplasmic side of the cortical actin layer in the mouse oocyte. The distribution of Rho A was polarized with the highest concentration occurring in the microvillar zone of the cortex. Most of the larger Rho A aggregates were localized below actin fenestrae and exhibited no physical interaction with β-catenin. Rho A has been implicated in cortical granule migration during oocyte maturation in sea urchins (Covián-Nares et al., 2004), and one might speculate that it could direct movement of cortical granules to fenestrae in the mouse oocyte. Given the complexity of the cortical actin layer, it is appropriate to propose functional differences between fenestrae and interstitial regions of cortex and between the inner and outer sides of the actin layer.

## Summary

The present study reveals that β-catenin and Rho A are recruited to the sperm binding site at about the same stage of fertilization as PTK2B and that β-catenin recruitment is dependent, at least in part, on the presence of PTK2B and CD9. We propose that CD9 enables the oocyte to respond to sperm binding by activation of PTK2B which could enable β-catenin to promote actin remodeling events that induce enlargement of microvilli already bound to the sperm head. The resulting enhancement of surface contact between sperm and egg PMs would potentially favor tighter sperm binding as proposed (Takezawa et al., 2011), leading ultimately to fusion competent sites that could lead to gamete fusion (Jégou et al., 2011). Future live cell imaging studies could determine whether gamete fusion occurs at fenestrae or at interstitial regions. Other results presented here also show that the cortical actin layer within the microvillar zone is divided into fenestrae where the actin layer is attenuated, and the surrounding interstitial region which is thicker. This data raises the possibility that the PM may also have different physical or compositional properties depending on whether it overlies the interstitial regions or the fenestrae. Future studies with live oocytes could reveal differences in membrane fluidity between fenestrae and interstitial regions and how the fenestra changes during sperm incorporation. The significance of the above findings is not readily apparent, but it is hoped that the results can stimulate additional studies which reveal how the cortical actin layer functions globally, as well as locally at the sperm binding site.

## Data Availability

Images used for the figures in this study are available at “http://www.cellimagelibrary.org.” Datasets used in this study are available from the corresponding author on reasonable request.
