# Supplementary figures and images for: Signaling Proteins Recruited to the Sperm Binding Site: Role of β-Catenin and Rho A
Source: Front Cell Dev Biol. 2022 May 13;10:886664. doi: 10.3389/fcell.2022.886664 (PMC9136404; doi:10.3389/fcell.2022.886664)

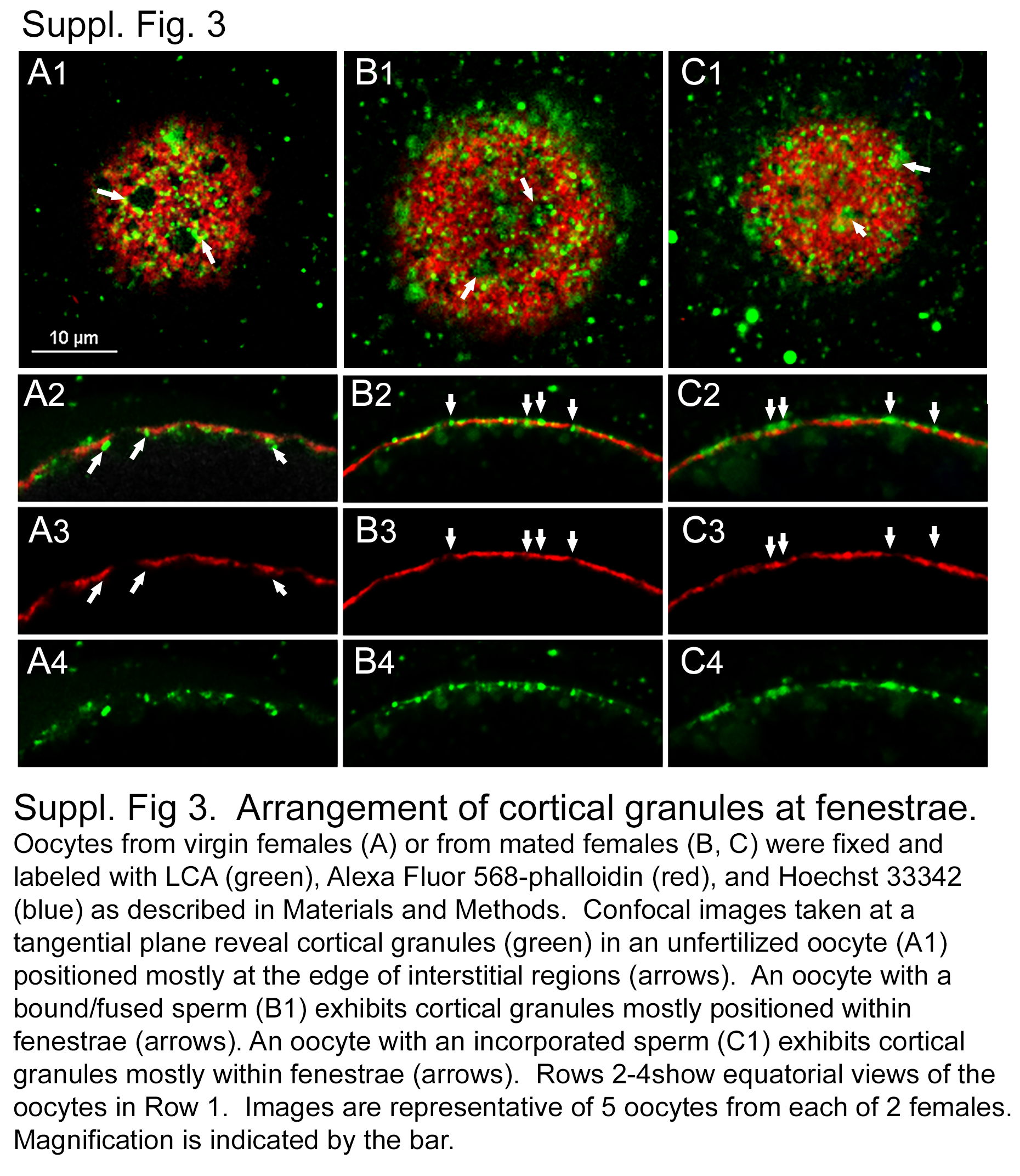

Supplement: Supplementary file 1 [file Image3.TIF]

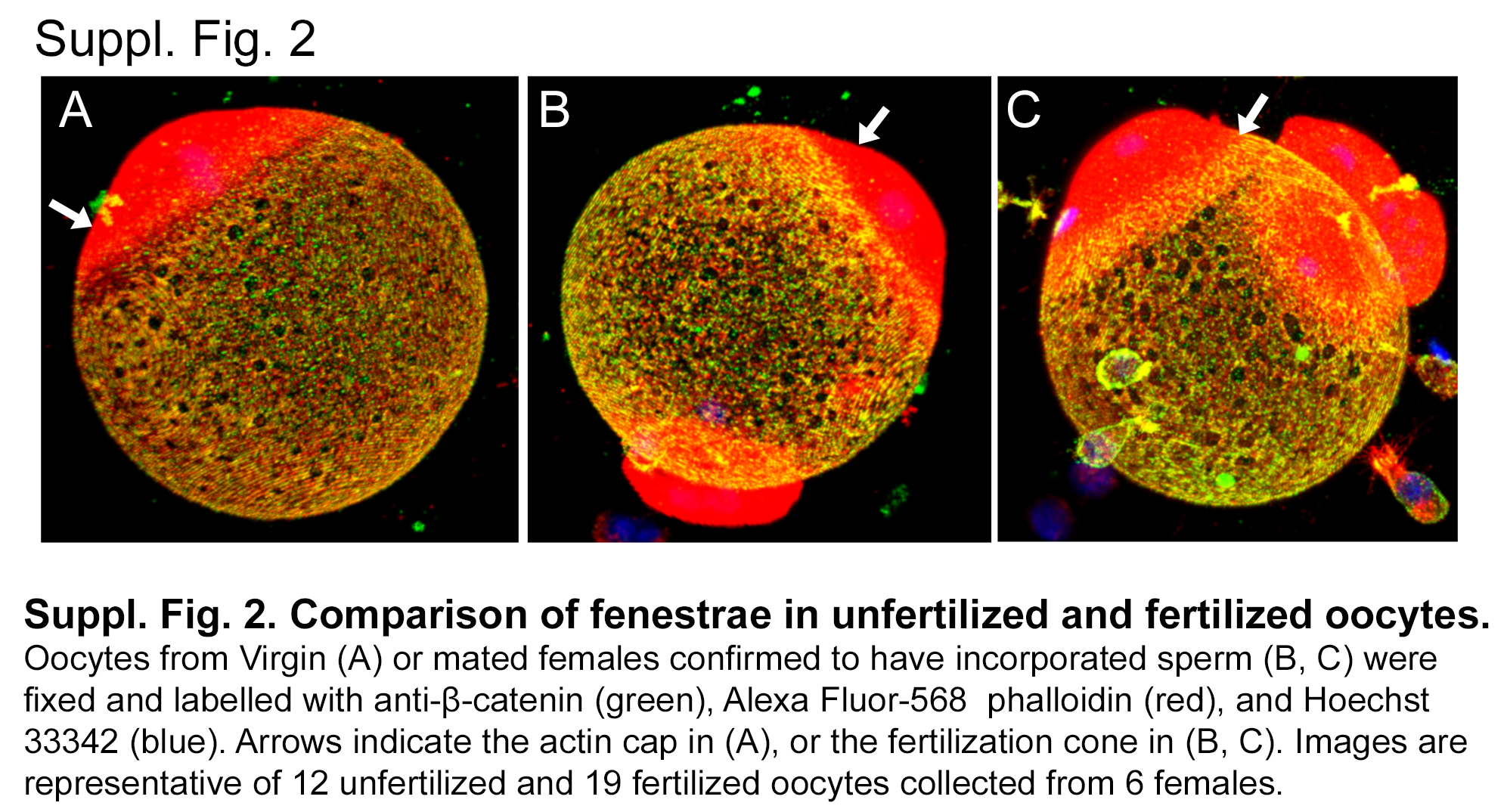

Supplement: Supplementary file 2 [file Image2.TIF]

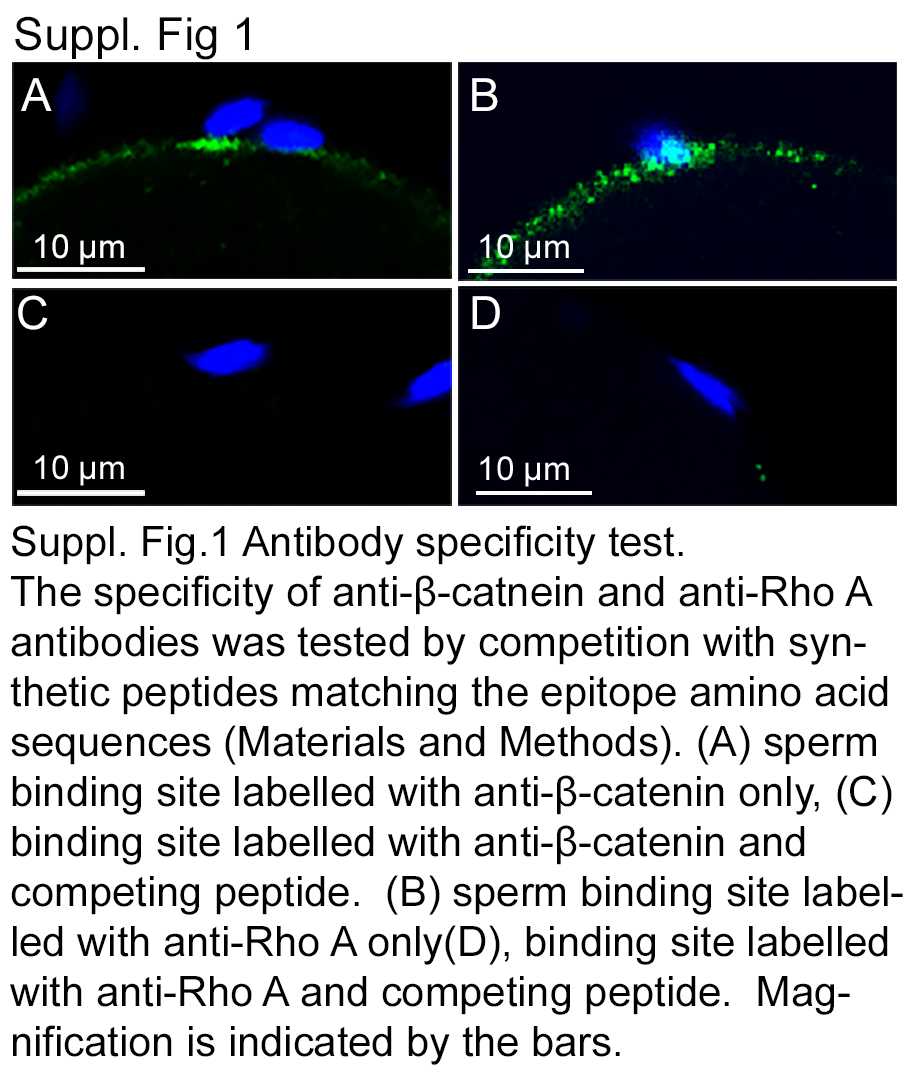

Supplement: Supplementary file 3 [file Image1.TIF]
